# Getting to Zero New Tuberculosis Infections: Insights From the National Institutes of Health/US Centers for Disease Control and Prevention/Bill & Melinda Gates Foundation Workshop on Research Needs for Halting Tuberculosis Transmission

**DOI:** 10.1093/infdis/jix311

**Published:** 2017-11-03

**Authors:** N Sarita Shah, Peter Kim, Bavesh Davandra Kana, Roxana Rustomjee

**Affiliations:** 1 Division of Global HIV and Tuberculosis, US Centers for Disease Control and Prevention, Atlanta, Georgia;; 2 Office of AIDS Research, and; 3 Tuberculosis Clinical Research Branch, Division of AIDS, National Institutes of Health, Rockville, Maryland;; 4 Department of Science and Technology-National Research Foundation (DST-NRF) Centre of Excellence for Biomedical TB Research, Faculty of Health Sciences, University of the Witwatersrand, National Health Laboratory Service, Johannesburg, and; 5 Medical Research Center-Center for the AIDS Programme of Research in South Africa (MRC-CAPRISA) HIV-TB Pathogenesis and Treatment Research Unit, Centre for the AIDS Programme of Research in South Africa, CAPRISA, Durban, South Africa

Tuberculosis caused an estimated 1.4 million deaths in 2015 and now ranks as the leading infectious disease cause of mortality in the world [1]. An additional 1.7 billion people are currently infected with *Mycobacterium tuberculosis* and are at risk of developing active tuberculosis disease. The challenge to eliminate tuberculosis has never been more relevant and urgent. Unfortunately, efforts to bring this global epidemic under control have been hampered by inadequate understanding of the epidemiology, biology, and effective interventions that directly address tuberculosis transmission. Identifying the key drivers of transmission and appending interventions has thus far remained unattainable in many high-burden tuberculosis settings. The large reservoir of subclinical infections, the human immunodeficiency virus (HIV) coepidemic, and a rise in drug resistance make public health efforts to battle this disease complex. For example, in southern Africa, high rates of HIV infection have served as key drivers of the tuberculosis epidemic and have created a complex clinical situation with unique difficulties in diagnosis and tracking infectious individuals; these limitations have seriously hampered tuberculosis elimination in the region [1]. In the United States, intermittent outbreaks of tuberculosis continue to occur despite utilization of modern prevention interventions in a well-funded healthcare system [2]. Globally, the modest declines (of 1.5%) in tuberculosis incidence between 2014 and 2015 fall markedly short of the targeted 4%–5% annual decline that is needed to achieve the 2020 milestones for the global End TB Strategy [3]. Of particular concern, the massive rollouts of new interventions targeted at individual patients, such as molecular diagnostics, preventative therapy, and antiretroviral treatment, and infrastructure investments in renovating healthcare facilities to reduce airborne disease spread have not resulted in sufficient epidemiological impact to meet global goals in reducing tuberculosis incidence [4, 5]. This highlights the need for sharply focused, evidence-led interventions and strategies tailored to individuals and communities, which are complemented by an ambitious implementation science agenda that rapidly translates new research findings to the field. Central to this process should be a paradigm shift toward limiting transmission with improved efficiency and effectiveness and the incorporation of strategies for overcoming the stigma and discrimination that continues to dilute the uptake and utilization of health services.

The issue of transmission is at the core of nearly every infectious disease epidemic and begins with understanding among whom, where, and how a disease is being spread and how this spread is amplified by risk factors and drivers at an individual and community level. Conventionally, we know tuberculosis is spread from person to person through airborne transmission of tubercle bacteria via droplet nuclei expelled by an infectious person in close proximity of a susceptible individual. Sufficient sharing of air space is important for a successful chain of transmission. Only a small proportion of individuals infected with *M. tuberculosis* develop active TB disease; the large majority are able to contain or eliminate infection. Individuals with compromised immunity (eg, HIV, diabetes, older age, malnutrition) or underlying lung disease are at high risk of developing active tuberculosis disease. The development of active tuberculosis, which results in lung damage and respiratory insufficiency, creates the opportunity for transmission of infection to other susceptible individuals. A critical factor driving tuberculosis transmission is the availability of a large pool of individuals who can be infected; vaccination provides a marginal benefit to the adolescent and adult population. Furthermore, individuals who previously had tuberculosis infection or tuberculosis disease and have been cured can become reinfected, particularly in high-burden settings where they are repeatedly re-exposed to bacteria.

In March 2016, the National Institutes of Health (NIH), US Centers for Disease Control and Prevention (CDC), and the Bill & Melinda Gates Foundation convened a 2-day meeting of physicians, research scientists, individuals working in public health, and other stakeholders to discuss the current state of the art regarding tuberculosis transmission and what is needed to truly transform our approach to halting new infections. Moving beyond implementing existing tools—which have the potential to create impact in focused, strong programs—to the point of having game-changers that will redirect effort and resources was the main objective of the dialogue. The workshop took a holistic approach to transmission and served as a platform to catalyze ideas and crystallize a vision for elimination of tuberculosis by highlighting transmission-blocking strategies as the central component of the overall effort directed at this deadly disease.

The articles in this supplement present the 3 major thematic areas addressed in the workshop. First, who is infectious and who gets infected? Second, what are the factors that drive transmission in the community? Third, how should interventions be designed and evaluated for maximal impact? The supplement closes with a research roadmap that outlines priority areas for tuberculosis transmission science and some metrics for measuring progress toward those goals. Together, these articles provide a comprehensive review of the current state of tuberculosis transmission science and a vision for the way forward to inform future research. The presentations from the workshop are publically available at http://tbtw.auruminstitute.org/. A follow-up workshop was held in June 2017 in Cape Town, South Africa; it focused specifically on halting tuberculosis transmission in HIV-endemic settings, where the risk of tuberculosis infection and consequences of tuberculosis disease are most dire.

It is a profoundly sad reality that modern medicine is defined by the fact that a person dies of tuberculosis every 20 seconds. No longer can we ignore the growing menace of tuberculosis and the heartfelt plight of those suffering with this disease. We anticipate that this supplement will be an urgent call to action to address tuberculosis transmission with renewed vigor using a focused, multidimensional strategy that calls on the strengths of all disciplines. With this unified approach, we *will* see an end to tuberculosis.

## Notes

***Financial support.*** This publication and workshop have been funded in whole or in part with federal funds from the Division of AIDS, National Institute of Allergy and Infectious Disease, US National Institute of Health, Department of Health and Human Services under contract number HHSN272201100001G—Research Support Services for the Division of AIDS. The Bill & Melinda Gates Foundation provided funding for participant travel to the workshop. The US Centers for Disease Control and Prevention and the South African Medical Research Council cohosted the corresponding meeting. B. D. K. was supported by an International Early Career Scientist Award from the Howard Hughes Medical Institute and grants from the National Research Foundation of South Africa and the South African Medical Research Council. This work has been supported in part by the President’s Emergency Plan for AIDS Relief (PEPFAR) through the US Centers for Disease Control and Prevention and by the US National Institute of Allergy and Infectious Diseases, National Institutes of Health (R01AI089349).

***Supplement sponsorship***. This work is part of a supplement sponsored by the Centers for Disease Control and Prevention and the National Institutes of Health.

***Potential conflicts of interest.*** R. R. is contracted by Kelly Services, which receives federal funds through a government contract with the National Institutes of Health. All other authors report no potential conflicts of interest. All authors have submitted the ICMJE Form for Disclosure of Potential Conflicts of Interest. Conflicts that the editors consider relevant to the content of the manuscript have been disclosed.
